# Neurotropism of Salvarsan

**Published:** 1911-10

**Authors:** 


### Neurotropism of Salvarsan

The authors describe thirteen cases in which nervous disturbances followed the use of salvarsan. The first case exhibited symptoms of irritation of the meninges of the brain and spinal cord one month after the injection; a second injection caused all the symptoms to subside. The other cases showed, respectively, signs of basal meningitis with bilateral facial paralysis; basal meningitis with bilateral facial paralysis and involvement of the auditory nerves; bilateral otitis interna with optic neuritis on the right side and homonymous hemianopsia; facial paralysis; oculomotor paralysis; otitis interna of the left ear; optic neuritis with irritation of the cerebral and spinal meninges and left otitis interna; otitis interna of the left ear; alternating bilateral otitis interna; bilateral otitis interna; otitis interna with especial involvement of the vestibular nerve; otitis interna with Meniere's symptom-complex. All these patients except one were in the early stage of secondary syphilis, and the nervous symptoms appeared six or eight weeks after the injection of salvarsan.

These phenomena were not due, in the opinion of the authors, entirely to a neurotoxic action of salvarsan, but were the result of an inflammatory infiltration of nervous structures dependent upon the action of the spirochetes or their toxins and easily responsive to further treatment, recovery being the rule. They have seen four other syphilitics with involvement of the auditory nerve who had never been subjected to salvarsan treatment, but had taken only mercury. Nevertheless, the frequent occurrence of the nervous phenomena in association with salvarsan administration would indicate, according to the authors, a certain degree of affinity on the part of salvarsan for these nerves insufficient to cause a degenerative inflammation, but sufficient to induce localization of the syphilitic process in them. They advise, in consequence, early and vigorous mercurial treatment in these cases.—Geronne and Gutmann, The *Berliner klinische Wochenschrift*.—*Charlotte Medical Journal.*

---

If thou art worn and hard beset
With sorrows that thou wouldst forget;
If thou wouldst read a lesson, that would keep
Thy heart from fainting and thy soul from sleep,
Go to the woods and hills! No tears
Dim the sweet look that Nature wears.

